# Changes in Pectoral Muscle Volume During Subacute Period after Radiation Therapy for Breast Cancer: A Retrospective up to 4-year Follow-up Study

**DOI:** 10.1038/s41598-019-43163-0

**Published:** 2019-05-07

**Authors:** Anna Seo, Jong-Moon Hwang, Jong-Min Lee, Tae-Du Jung

**Affiliations:** 1 Lee Gil Ya Cancer and Diabetes Institute, Gachon University of Medicine and Science, Yeonsu-gu, Incheon Korea; 2 Department of Rehabilitation Medicine, Kyungpook National University Hospital, Daegu, Korea; 3 Department of Rehabilitation Medicine, School of Medicine, Kyungpook National University, Daegu, Korea; 4 Department of Radiology, Kyungpook National University School of Medicine, Daegu, Korea

**Keywords:** Breast cancer, Breast cancer, Skeletal muscle, Skeletal muscle, Breast cancer

## Abstract

Radiotherapy (RT) is an effective treatment for managing breast cancer patients with breast conserving surgery, but patients may experience radiation-induced shoulder problems. Even though the course of shoulder morbidity is unknown, pectoral muscle changes after radiotherapy can be a major cause of shoulder problems. Twenty-two patients treated with RT for unilateral breast cancer were included in the study. All patients underwent serial computed tomography (CT) imaging before and immediately after RT, as well as 2 months, 6 months, 2 years, and 3–4 years after RT. These CT scans were used to compare muscle volume changes. The pectoral muscle volume and muscle volume surrounding the scapular measurement was performed using 3D modelling after segmentation of the CT scans. In all patients, the pectoral muscle volume increased during the 2 months after RT, and there was continuous volume reduction from 2–48 months after RT. Changes in muscle volume ratio over time were analysed by repeated measure ANOVA and it was found that there was a significant change in the pectoral muscle volume (p < 0.001) from Just before RT and Immediately after RT at 2 month after RT. On the other hand, the changes in the muscle volume of the surrounding scapular were not significant.

## Introduction

Breast cancer is the most frequently diagnosed cancer in women^1^. In 2012 alone, approximately 1.7 million new breast cancer cases were diagnosed worldwide^2^. Radiotherapy (RT) is the standard treatment for over 90% of these patients^3^. RT after breast conserving surgery (BCS) is associated with a 22% reduction in the 10-year local recurrence rate^4^. RT may also increase long-term survival of patients who present with early-stage breast cancer^5–7^.

The recent introduction of new surgical approaches, such as sentinel lymph node biopsy (SLNB), and a reduction of RT to the axilla are expected to result in a decrease, but not the complete elimination, of shoulder morbidity among breast cancer patients^8–13^. Johansen *et al*.^14^ recently evaluated impaired shoulder motion after RT and surgery for breast cancer. They stated that damage to the pectoralis major muscle was the most important factor in the development of this complication. Pectoral muscle length is believed to have an important effect on resting shoulder girdle alignment^15^. Decreasing the size of the pectoral muscle may affect the ability of the patient to reach upward as this muscle extensibility is required to disengage the humeral head from the glenoid cavity at the end of humeral elevation^16^.

Muscle changes were mostly observed after 3 years at the latest after RT^17,18^. In breast cancer, clinical findings have been reported on muscle morbidity of the pectoralis major^17,19^. However, there are no studies on the early changes in monthly pectoral muscle volume for the 6 months immediately after RT and the cause of shoulder problems in breast cancer patients. Moreover, previous studies did not exclude the adverse effects of RT on the axillary site, which may play an important role in shoulder morbidity.

Deutsch *et al*. represents the limited range of motion of the shoulder and the possibility of increased shoulder pain in patients who were followed up for 6 months after induction cancer resection and who received radiotherapy after breast cancer surgery^20^. Janine T. Hidding *et al*.^21^ found level 1 evidence for axillary lymph node dissection [ALND], and concurrent radiotherapy and chemotherapy as risk factors for reduced muscle strength. also they found level 2 evidence for sentinel node biopsy [SNB], radiotherapy to the chest wall and radiotherapy to the axilla and chest as risk factors for reduced muscle strength. The extent to which factors such as surgery, radiotherapy and patient characteristics affect of limited shoulder motion and shoulder pain is not well documented.

The purpose of this study is to find clues to the causes of shoulder pain in patients with breast cancer. We hypothesized that it was a problem of shoulder-related muscles affected by RT for reasons of shoulder pain. We excluded as many as possible causes of shoulder problems(surgery method, RT area ets) when treating patients with breast cancer and noted the problem caused by pectoral muscle affected by RT. Using 3D modelling based on computed tomography (CT) imaging after RT, we examined the effects of radiation therapy, commonly used in breast cancer patients, on muscles through a timely change in pectoral muscles volume.

## Methods

### Ethics statement

We retrospectively reviewed patient CT scans between 2011 and 2017 after obtaining approval from the Institutional Review Board of Kyungpook National University Hospital (No. KNUH_2017-09-11) and approved the research protocol of the present study, and all methods were carried out in accordance with the relevant guidelines and regulations. The IRB decided that the acquisition of informed consent from 22 patients was not required according to the guideline because this was a retrospective observational study.

### Participants

Table 1 shows the inclusion and exclusion criteria of the experimental group. A total of twenty-two patients were included in the study. All patients underwent BCS and SLNB and had received RT which was applied only to the trunk region, while the axillary area was excluded. Patients who underwent axillary lymph node dissection or who received RT to the axillary site were excluded.

**Table 1:** Inclusion and exclusion criteria.

Inclusion criteria	Exclusion criteria
Unilateral carcinoma of the breast Treatment protocols*	
(1) BCS + SLNB + radiotherapy to the trunk	(1) No radiotherapy
(2) BCS + SLNB + radiotherapy to the trunk + chemotherapy	(2) Radiotherapy to axillary
(3) BCS + SLNB + radiotherapy to the trunk + hormone therapy	(3) BCS with ALND
(4) BCS + SLNB + radiotherapy to the trunk + chemotherapy + hormone therapy	(4) Mastectomy
	(5) Reconstructive surgery
	(6) Current or previous history of shoulder problem

*BCS*, breast conservation surgery; *SLNB*, sentinel lymph node biopsy;

*ALND*, axillary lymph node dissection.

An *a priori* sample size calculation was performed after the first 5 patients were analysed (partial eta squared = 0.861). Using a repeated measures ANOVA, a significant within-subject interaction (alpha = 0.05 level) could be detected with 80% power using 10 patients.

Twenty-two women meeting the inclusion and exclusion criteria (Table 1) consented to take part in this study. The patients had a mean age of 46.78 ± 6.99 years (range: 31–60 years). Fifteen patients were treated on the right side and 7 patients were treated on the left side of the thorax. Breast cancer staging was I for 20 patients and II for 2 patients (Table 2). We observed the period from the time immediately after radiation therapy to 3–4 years post-treatment.

**Table 2:** Subject demographics and characteristics.

Case	Affected side	Age	The period between surgery and RT, days	Stage of breast cancer (IDC)	Treatment	Number of lymph nodes sampled	Hand dominance
1	Lt	45	216	II	BCS + SLNB, RT + CTx + HTx	1	Rt
2	Rt	60	20	I	BCS + SLNB, RT + HTx	4	Rt
3	Lt	46	119	I	BCS + SLNB, RT + CTx + HTx	3	Rt
4	Rt	55	113	I	BCS + SLNB, RT + CTX	1	Lt
5	Lt	55	26	I	BCS + SLNB, RT	3	Rt
6	Lt	51	212	I	BCS + SLNB, RT + CTx + HTx	6	Rt
7	Rt	50	206	I	BCS + SLNB, RT + CTx + HTx	4	Rt
8	Rt	50	129	I	BCS + SLNB, RT + CTx + HTx	1	Rt
9	Rt	50	52	I	BCS + SLNB, RT + HTx	3	Rt
10	Rt	40	121	I	BCS + SLNB, RT + CTx	3	Rt
11	Rt	46	31	II	BCS + SLNB, RT + HTx	6	Rt
12	Lt	34	124	I	BCS + SLNB, RT + CTx	1	Rt
13	Rt	47	29	I	BCS + SLNB, RT + HTx	1	Rt
14	Rt	44	33	I	BCS + SLNB, RT + HTx	5	Rt
15	Rt	31	125	I	BCS + SLNB, RT + CTx + HTx	6	Rt
16	Lt	46	36	I	BCS + SLNB, RT + HTx	1	Rt
17	Rt	40	120	I	BCS + SLNB, RT + CTx	2	Rt
18	Rt	37	138	I	BCS + SLNB, RT + CTx + HTx	3	Rt
19	Rt	43	28	I	BCS + SLNB, RT + HTx	1	Rt
20	Lt	49	107	I	BCS + SLNB, RT + CTx + HTx	3	Rt
21	Rt	53	217	I	BCS + SLNB, RT + CTx	3	Rt
22	Rt	45	126	I	BCS + SLNB, RT + CTx	4	Rt

*IDC* invasive ductal carcinoma, *BCS* breast conservation sugery, *SLNB* sentinel lymph node biopsy, *RT* radiotherapy to the trunk, *CTx* chemotherapy, *HTx* hormone therapy, *CT* computed tomograph.

Six CT scans were obtained for clinical care. Two scans were for RT planning and the remaining 4 were follow-up CT scans after RT. In all scans, the patient’s arms were fully abducted, with the patient’s elbows flexed and hands placed behind the head. CT scans were performed just before RT, immediately after RT, and 2 months, 6 months, 2 years, and 3–4 years after RT, using the same posture in the supine position.

### Radiotherapy

All patients received CT scans with a slice thickness of 5-mm in the supine position with overhead of both arms. Thereafter, the CT data was transferred to a treatment planning system (Eclipse; Varian Medical Systems Inc., Palo Alto, CA, USA). The clinical target volume (CTV) was defined as the total breast tissue, including the glandular breast and circumferential soft tissue. The target volume was defined as a 5 mm margin to the CTV reference in all directions except for the outer skin margin. The treatment plan was implemented with two tangential fields for breast irradiation. The upper margin of the irradiation field was at the head of the clavicle and the lower margin was 2 cm below the intra-mammary fold^22^. The planned dose was normalized to the dose at a point 0.5–2.0 cm on the chest wall-lung interface. The the wedge angles of the tangential beam and the weight of the field were optimized to obtain a uniform dose distribution.

A dose calculation algorithm was used to determine the inhomogeneity correction using the Clarkson method. In principle, the prescribed dose of tangential irradiation was 50 Gy in 28 fractions over the course of 5 weeks in 22 patients. In addition, a radiation boost was given to 22 patients, at a dose of 10 Gy in 5 fractions to the trunk region only.

### Muscle volume estimation

**Figure 1:**
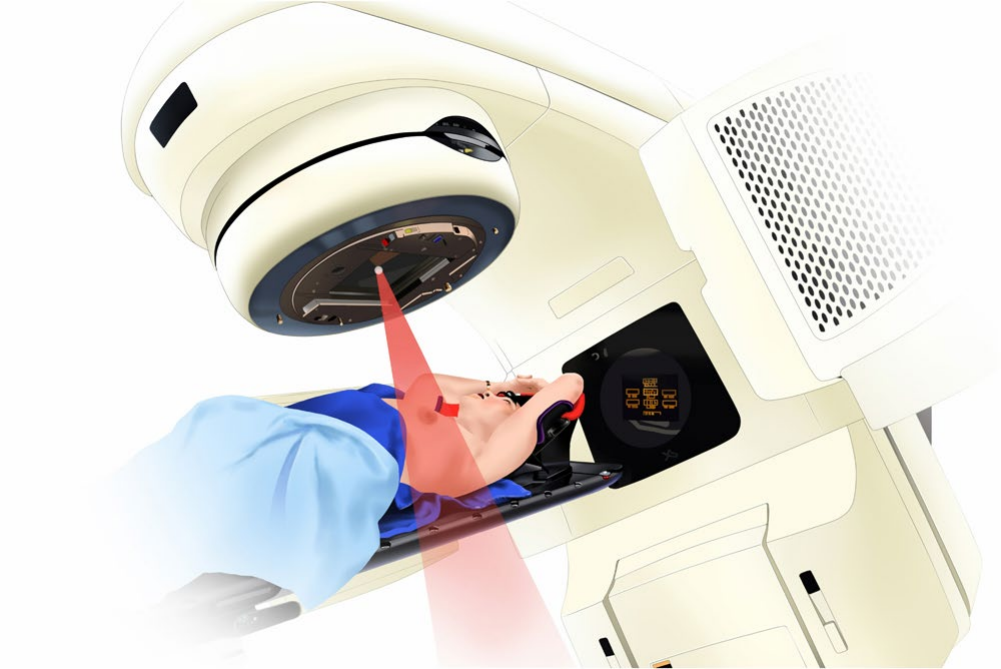
The FOV (Field Of View) concept in radiotherapy after breast cancer surgery.

The Radiation was exposed for radiation therapy (Fig. 1) after breast cancer surgery and the volume of the affected breast areas (pectoralis major and pectoralis minor). We compare changes of the ratio of muscle after radiotherapy and confirm the effect. Three-dimensional (3D) muscle volume was measured to analyze changes in the volume of the breast muscle (pectoralis major and pectoralis minor) of patients who received radiation therapy after breast cancer surgery.

The 3D volume of the pectoralis major muscle and pectoralis minor muscle was measured 6 times on the images just before treatment(1), immediately after treatment(2), 2 months(3), 6 months(4), 2 years(5), and 3~4 years(6) after treatment. Also in order to measure the volume change of the muscle that is not affected by radiation, the volume of muscle(supraspinatous, subscapularis, infraspinatous, teres minor) surrounding the scapula was measured and the ratio of volume change was analyzed.

The data used in this study are CT images that were followed up to 4 years postoperatively. Since it takes 6 times for a considerable period of time, so there may be a difference in image between shots even for the same patient.

In order to overcome the error that the patient’s posture may change at upon acquisition time, we used a method to keep the same position by taking a posture that supports the head of the wrist by hand at every acquisition. In addition, the area to measure the volume is not the data of a specific point, but specifies the range (VOI: Volume Of Interest) to specify and divide the area having the same rule, and the entire muscle volume exists within the range area was measured. Therefore, even if the posture changes a bit at the acquition time, the method of overcoming the error at the time of shooting is used by measuring the range of the volume with the same rule.

We performed segmentation of three step to measure the 3D volume of the breast muscle affected by radiotherapy and reconstruct the segmented results (2D mask) to 3D model.

Step 1. **Whole muscle and bone:** Semi-automatic Segmentation of the whole muscle areas including bone areas and Bone areas with HU (Hounsfield Unit) value

Step 2. **Muscle areas only:** Perform a Boolean operation that subtracts the bone region from the muscle region as a result of step 1 to remove overlapping areas of muscle and bone area

**Figure 2:**
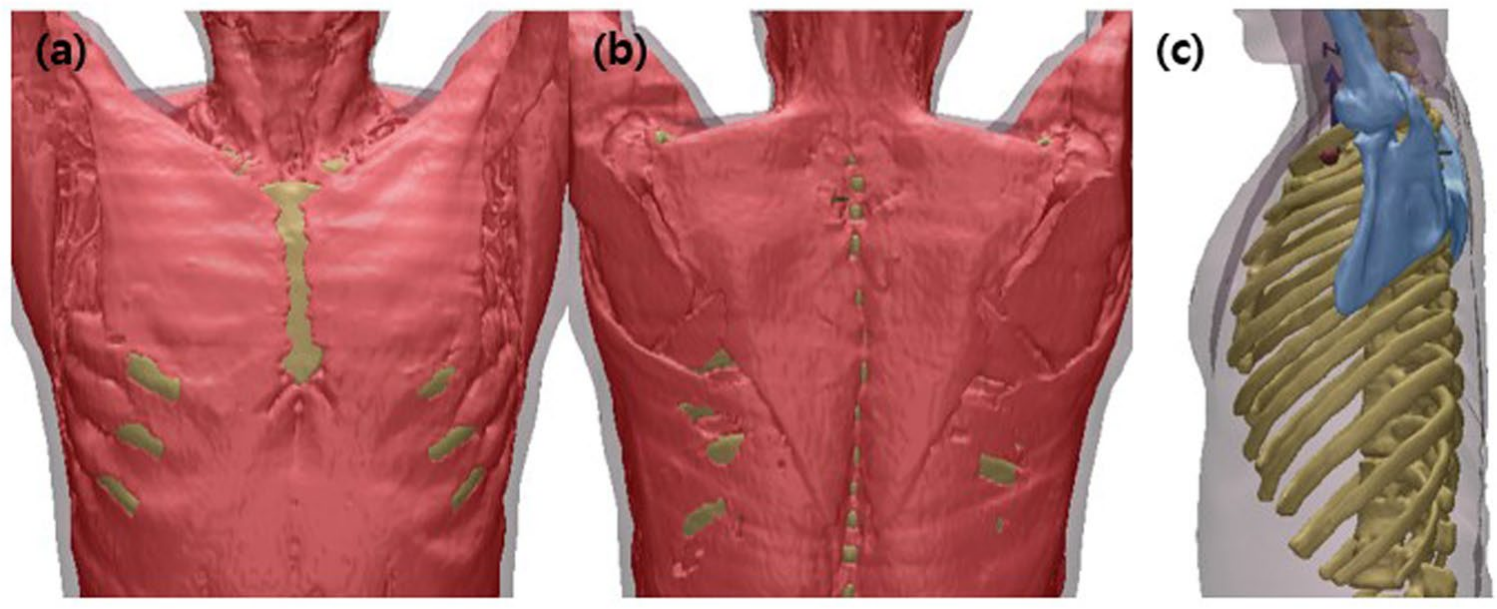
Segmentation of Whole muscle and bone by HU(Hounsfield Unit) value: (**a**) anterior view, (**b**)posterior view, (**c**) Left view.

Step 3. **Breast muscle:** Separation of the pectoralis major muscle and pectoralis minor muscle after setting the VOI (Volume Of Interest) in 3D Space with the rules shown in Fig. 2.

We use the semi-automatic segmentation method as much as possible to prevent the error of the muscle boundary caused by the person who manipulates as much as possible caused by manual division method. The Mimics v20 medical imaging processing software (Materialise, Leuven, Belgium) was used to segmentation of breast muscle and reconstucted three-dimensional model.

The HU(Hounsfield Unit) values used in the first phase of the semi-automatic segmentation of muscles and bones are expressed in intensity value, and the segmentation process is carried out by dividing the range of values including both the muscle and the bone domain (Fig. 2a–c).

In Step 2, to distinguish overlapping areas of the total muscle and bone regions divided by Step 1, a Boolean operation is performed to remove bone areas from the muscle region.

**Figure 3:**
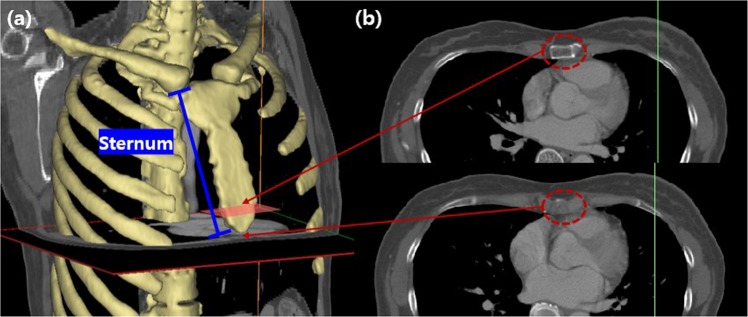
A range of breast muscle(from the top to the bottom of the sternum) (**a**) 3D Chest model(bone) and The range of sternum including the pectoralis major and minor muscles(blue line) (**b**) 2D image corresponding to each location within the 3D model.

The last step in the process of splitting step 3, reconstructs the 3D breast muscle after dividing only the pectoralis major muscle and pectoralis minor muscle regions of the breast muscle based on the sternum in the entire muscle region resulting from the step 2 process. Range of breast muscle was defined from the top to the bottom (xiphisternal junction line) of the sternum in this paper, with respect to the horizon for a given measurement range (Fig. 3a, blue line).

We did not include three muscles in the xiphoid region, which shows an unknown HU value in CT images, in order to apply semi-automatic segmentation method rather than a manual division of individual segmentation results.

**Figure 4:**
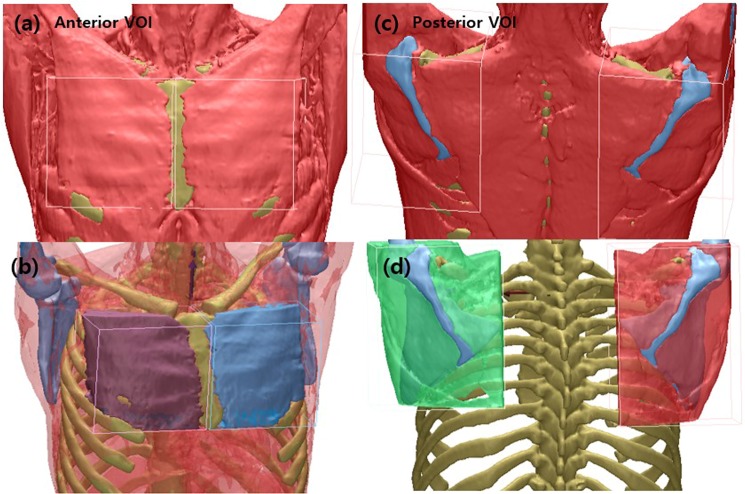
Setting of VOI(Volume Of Interest) and Result: (**a**) Anterior View for segmentation of Breast muscle(pectoralis major and pectoralis minor) in the 3D space, (**b**) result of separated breast muscle (**c**) Posterior View for segmentation of muscles surrounding the scapular, (**d**) result of separated muscles of back that surrounding the scapular.

In order to analyze the muscles affected by the radiation by applying the range designation rule, the left and right breast muscles were divided by specifying the VOI in the three-dimensional space (Fig. 4a). In order to analyze the changes of the muscles that are not affected by radiation therapy, we performed the three-step process applied to the division of the breast muscle to divide the muscular region by designating VOI (Volume Of Interest) in the muscle region containing the scapula 4-c) 3D model was created (Fig. 4d). Looking at the shoulder girdle muscle corresponding to this area on the basis of the scapular bone, the medial border of scapular includes the lower fibers of trapezius, rhomboideus major and minor muscle partly. With the scapular bone as a guide, lateral border contains teres minor, major, deltoid and latissimus dorsi muscle, superior border includes the upper fibers of trapezius, supraspinatus muscle part. And the muscles placed on the scapular bone include infraspinatus, supraspinatus and deltoid muscle part.

In a very rare case, when CT scan was performed in a tilted position, after setp1 was performed, the clavicle and sternum were rotated so that the direction of the clavicle and sternum was perpendicular to each other in the three-dimensional space, and then step 3 was performed. There is a difference in image depending on the photographing posture and Individuals do not have the same weight, height, and breast size, so absolute value comparison of the muscle volume is meaningless. In order to analyze the volume change of the operated region, we used the method of calculating the ratio of the left and right breast muscle(pectoralis major and pectoralis minor) using Equation 1 to measure the volume of the 3D muscle model generated through the segmentation process (Fig. 4c,d). In the three-dimensional muscle volume measurement method, a method of comparing the volume of the opposite direction breast based on the volume of the breast surgically operated with only the operation of one of the left or right breasts was used. In other words, the volume of the person who operated was converted to a numerator, and the volume of the other side that had not been operated was converted into the denominator (Equation 1). For example, the value of the patient who underwent surgery on the right side of the breast would be denoted by: (right breast muscle volume)/(left breast muscle volume).

$$M{V}_{ratio}=\frac{RT{B}_{-}V}{URT{B}_{-}V}\,\,\begin{array}{l}MVratio:{\rm{The}}\,{\rm{ratio}}\,{\rm{of}}\,{\rm{one}}\,{\rm{breast}}\,{\rm{surgery}},\\ RT{B}_{-}V:{\rm{The}}\,{\rm{operated}}\,{\rm{side}}({\rm{Radiation}}\,{\rm{therapy}}),\\ URT{B}_{-}V:{\rm{Unoperated}}\,{\rm{side}}({\rm{Untreated}})\end{array}$$

Equation. 1 the ratio of the left and right breast muscle (pectoralis major and pectoralis minor).

### Statistical methods

A repeated-measure the analysis of variance (ANOVA) with sequence and trial as the independent variables was used to evaluate performance changes across repeated measurements. Bonferroni post-hoc tests were conducted if a significant interaction effect was found. A value of p < 0.05 was considered to be statistically significant. The statistical analyses were performed with SPSS version 20.0 (SPSS Inc., Chicago, USA) and the significance level was set at 0.05. Muscle volume estimation were graded by the consensus of two physiatrists blinded to other clinical information. The intra-class correlation coefficient (ICC) model of the volume measurement was calculated to test inter-observer reliability(two-way mixed model, single measures). An ICC value > 0.80 was considered very good; between 0.61 and 0.80, good; 0.41–0.60, moderate; 0.21–0.40, fair; < 0.21, poor. The consistency of other items was evaluated using Cohen’s kappa.

## Results

Demographic and medical details are shown in Table 2, including the details about age, affected side, hand dominance, stage, number of lymph nodes involved, and treatment protocol.

Inter-observer reliability was robust for muscle volume measurements using CT scans. The value of ICC(95% CI) was 0.896(0.830–0.937). The results of the continuous volumetric measurements after the RT are presented in Tables 3 and 4. Table 3 shows the ratio of affected and unaffected pectoral muscle volume using CT scan and Table 4 shows the ratio of muscle volume changes in specific areas around the scapular. Table 5 is Analysis of changes in muscle volume ratio over time with repeated measures ANOVA in pectoral muscle volume and separated muscles of back that surrounding the scapular. In all patients, the pectoral muscle volume tended to increase during the 2 months after RT, and then reduced in volume from 2 months to 4 years after RT (Table 3). Most of the increase in muscle volume was characteristic for the 2 months after RT, and the decrease in volume was mostly observed between 2 and 6 months after RT or during 6 months to 2 years after RT.

**Table 3:** Pectoral muscle volume ratio of affected and unaffected using CT scan.

Case	Just before RT	Immediately after RT	2 month after RT	6 month after RT	2 years after RT	3~4 years after RT
1	1.181	1.222	1.320	1.289	1.092	1.085
2	1.046	1.039	1.417	1.018	0.883	0.856
3	1.156	1.167	1.473	1.198	0.952	0.844
4	0.962	0.978	1.280	1.153	0.942	1.020
5	1.027	1.100	1.143	0.987	0.701	0.967
6	0.983	0.927	1.123	1.103	0.869	0.861
7	1.017	0.954	1.127	1.119	0.999	0.908
8	0.857	0.972	1.235	0.916	0.934	0.975
9	0.972	0.993	0.980	0.981	0.885	0.868
10	0.971	0.944	1.156	1.092	0.926	0.913
11	0.968	1.056	1.197	1.151	0.939	0.809
12	1.113	1.086	1.204	1.061	0.842	0.858
13	0.911	0.933	0.980	0.852	0.875	0.928
14	0.906	0.948	1.096	0.949	0.914	0.738
15	1.145	1.082	1.133	1.098	0.864	0.918
16	1.033	1.003	1.265	1.004	1.083	0.916
17	1.091	1.109	1.199	1.087	0.985	0.911
18	1.097	1.060	1.215	1.124	0.958	0.969
19	1.138	1.123	1.278	0.943	0.814	0.867
20	0.972	1.081	1.100	1.016	0.889	0.915
21	0.812	0.997	0.992	0.941	0.776	0.798
22	0.988	0.982	1.114	1.037	0.911	0.943
Mean ± SD	1.016 ± 0.099	1.034 ± 0.081	1.183 ± 0.127	1.051 ± 0.103	0.911 ± 0.089	0.903 ± 0.076

*RT* radiotherapy to the trunk, *CT* computed tomograph.

SD: standard deviation.

F value and p value for repeated measures ANOVA are 37.903 and < 0.001, respectively (degree of freedom = 5, sphericity assumed).

**Table 4:** Muscles surrounding the scapular volume ratio of affected and unaffected using CT scan.

Case	Just before RT	Immediately after RT	2 month after RT	6 month after RT	2 years after RT	3~4 years after RT
1	1.145	1.112	1.147	1.328	1.218	1.244
2	1.090	1.058	1.123	1.117	1.036	1.147
3	1.129	1.027	1.072	1.074	0.979	0.822
4	1.053	1.058	1.189	1.214	1.140	1.107
5	0.989	1.032	1.012	0.990	0.924	0.903
6	0.962	0.979	1.006	0.955	1.018	0.983
7	1.114	1.105	1.049	1.005	1.102	0.820
8	1.122	1.104	1.067	1.089	1.032	1.084
9	1.010	1.006	1.011	1.124	1.098	0.936
10	1.145	1.084	1.030	1.009	0.950	1.064
11	0.996	1.050	0.973	1.040	0.998	1.021
12	1.043	0.925	0.918	0.977	0.933	0.879
13	0.910	0.970	0.980	1.055	1.178	1.142
14	1.060	1.069	1.056	0.966	0.998	1.116
15	1.082	1.001	1.000	1.078	1.137	1.112
16	0.993	0.872	0.864	0.906	1.043	0.910
17	1.102	1.005	0.968	0.886	0.894	0.972
18	1.074	1.045	1.079	1.164	1.092	1.077
19	1.109	1.006	0.989	1.086	1.045	0.994
20	0.896	0.895	0.952	0.906	1.059	0.890
21	1.011	0.992	0.980	1.055	1.070	1.092
22	1.102	1.010	1.000	0.984	1.073	1.095
Mean ± SD	1.052 ± 0.072	1.018 ± 0.064	1.021 ± 0.074	1.046 ± 0.105	1.046 ± 0.823	1.019 ± 0.115

*RT* radiotherapy to the trunk, *CT* computed tomograph.

SD: standard deviation.

F value and p value for repeated measures ANOVA are 1.184 and 0.322, respectively (degree of freedom = 5, sphericity assumed).

**Table 5:** Post-hoc analysis of pectoral muscle volume ratio.

Test group	Difference of Mean	Standard Error	p value	p value (adjusted by Bonferroni)
Just before RT - Immediately after RT	0.01864	0.01356	0.184	>0.999
Immediately after RT - 2 month after RT	0.14868	0.02273	<0.001*	<0.001*
2 month after RT - 6 month after RT	−0.13218	0.02447	<0.001*	<0.001*
6 month after RT - 2 years after RT	−0.14027	0.0201	<0.001*	<0.001*
2 years after RT - 3~4 years after RT	−0.00755	0.02032	0.714	>0.999

RT radiotherapy to the trunk, CT computed tomograph.

*p < 0.05.

Changes in muscle volume ratio over time were analyzed by repeated measure ANOVA and it was found that there was a significant change in the pectoral muscle volume (p < 0.001). On the other hand, the changes in the muscle volume of the surrounding scapular were not significant. As a result of the Bonferroni post hoc test, it was found that the pectoral muscle volume increased significantly from Immediately after RT at 2 month after RT, and decreased significantly from 2 month after RT at 6 month after RT and from 6 month after RT at 2 years after RT (Table 5). On the other hand, Bonferroni post-hoc analysis of muscles surrounding the scapular volume ratio was not significant(Table 6).

**Table 6:** Post-hoc analysis of muscles surrounding the scapular volume ratio.

Test group	Difference of Mean	Standard Error	p value	p value (adjusted by Bonferroni)
Just before RT - Immediately after RT	−0.03327	0.01194	0.011	0.165
Immediately after RT - 2 month after RT	0.00273	0.00994	0.787	>0.999
2 month after RT - 6 month after RT	0.02468	0.01469	0.108	>0.999
6 month after RT - 2 years after RT	0.00041	0.01734	0.981	>0.999
2 years after RT - 3~4 years after RT	−0.02759	0.02185	0.22	>0.999

RT radiotherapy to the trunk, CT computed tomograph.

*p < 0.05.

## Discussion

In this study, we observed changes in pectoral muscle placed in the irradiated field up to 4 -year after RT for all breast cancer patients underwent BCS with SLNB and received RT applied only to the trunk region except for axillary area. Patients were required to perform 3D modeling after segmentation of the 2D image (CT) and to measure the volume of the 3D reconstructed model. In all patients, the pectoral muscle volume tended to increase for approximately 2 months after RT, and then decreased over the years after peaking. The increase in muscle volume for 2 months after RT was thought to be oedema caused by RT. There was no study of subacute phase after RT for this volume change.

We analyzed the volume changes of the breast area muscles affected by postoperative radiation therapy for breast cancer by dividing them into 6 volumes (2 months, 6 months, 2 years, 3~4 years before surgery) The changes were tracked. In this paper, we analyze the tracking change of the longterm period which was not covered in the existing paper, and measured the volume change with time in 3 - dimensional volume rather than 2 - dimensional data. In addition, the 3D volume change of the dorsal muscle was also measured for objective comparison between the affected muscle and the unaffected muscle.

Up to now, the results of research that breast cancer postoperative RT causes changes in muscle mass and influences shoulder movement and other areas is introduced. We analyzed how the volume change of the affected muscles affected the direct radiation in patients receiving such radiation therapy after breast cancer surgery^23–25^. Analyzing changes in muscle volume over the course of radiotherapy will help plan treatment cycles that minimize muscle loss or plan treatment options that set the range of radiation.

Generally, patients treated with RT after breast cancer surgery showed a decrease in the degree of internal rotation and adductor muscle strength on the side of breast surgery, compared with that of the opposite side^26^. Similarly, patients receiving the standardized RT protocol of 50 Gy to the breast and axilla show a significant decrease in shoulder range of motion and/or strength^26–28^. On the other hand, patients treated with RT after breast surgery showed a decrease in shoulder range of motion and/or strength, while patients with breast surgery alone without RT did not have lateral weakness^27^. In particular, atrophy of the pectoralis major and minor muscles has been demonstrated in breast cancer patients treated with standardized RT protocols^23^.

Since the pectoral muscles are typically in the RT field, it is not surprising they are highly influenced by the close proximity to the target breast tissue and anatomical location^29^. Reduction in the size of the pectoral muscles may affect a patient’s ability to reach overhead. Shamely *et al*.^23^ found that the pectoralis major and minor muscles decreased in size on the affected side in a series of 57 breast cancer patients from 6 months to 6 years post-surgery. The above study confirmed the change in muscle size by measuring the thickness of the pectoral muscles on magnetic resonance imaging (MRI), but did not provide any information within 6 months after RT. In another study, the authors observed changes in muscle size at 6 months intervals from 1 year before RT, to 3.5 years after RT, by measuring tissue thickness using MRI, but they did not confirm a change within 6 months after RT^30,31^. These studies considered long-term fibrosis, but did not mention early muscle changes within 6 months after RT, whereas in our study, early temporal changes of inflammation and oedema were indirectly confirmed by changes in muscle volume. The purpose of this study was to observe changes in specific muscles using 3D modelling based on CT scans before and after RT. These results suggest a potential relationship between muscle volume changes after radiation therapy at sub-acute time intervals and functional patient prognosis, but more research is needed to determine this effect, particularly using pathology.

This study has a few limitations. First, we did not consider the reduction of muscle volume caused by decreased motion of the arm and shoulder of the patient. Breast cancer patients who undergo surgery and RT have a tendency to limit their arm use to protect the arm and shoulder of the treated side^32^. This reduction in arm use led to a reduction in volume of the muscle associated with limb movement. If we considered this effect in our study, we would have likely seen a significant increase in muscle volume during the subacute phase caused by RT. Second, in our experiments, differences in muscle volume measurements between the physiatrists can affect the comparison of volume ratios. Third, the present study is that we did not clearly identify the cause of the increase in muscle volume in the subacute phase after RT. There are many factors that can cause volume increase of pectoral muscles during the subacute period such as vasculitis, tissue injury and denervation after RT^33–36^. Another limitation is the potential biasing of data based on differences in arm dominance. If the patient has an operation on his/her usual arm, there may be a difference in volume when he/she tries to use his or her familiar arm even after surgery.

In future studies it is necessary to determine treatment protocols by accurately identifying factors that cause RT-induced fibrosis or atrophy of pectoral muscles.

## Conclusion

Our finding emphasizes pectoral muscle changes after RT in breast cancer patients. The increase in volume at 2 months after RT can indirectly predict the sustained inflammatory response in the pectoral muscles that is present during the sub-acute phase after RT. These results provide clinicians and therapists with important information for the prevention of future shoulder problems caused by radiation therapy.

## References

[1] Yang TY, Chen ML, Li CC (2015). Effects of an aerobic exercise programme on fatigue for patients with breast cancer undergoing radiotherapy. J. Clin. Nurs..

[2] Ferlay J (2015). Cancer incidence and mortality worldwide: sources, methods and major patterns in GLOBOCAN 2012. Int. J. Cancer.

[3] Potthoff K (2013). Randomized controlled trial to evaluate the effects of progressive resistance training compared to progressive muscle relaxation in breast cancer patients undergoing adjuvant radiotherapy: the BEST study. BMC Cancer.

[4] Solin LJ (2010). Breast conservation treatment with radiation: an ongoing success story. J. Clin. Oncol..

[5] Clarke M (2005). Early Breast Cancer Trialists’ Collaborative Group (EBCTCG). Effects of radiotherapy and of differences in the extent of surgery for early breast cancer on local recurrence and 15-year survival: An overview of the randomised trials. Lancet.

[6] Steele GD, Jessup LM, Winchester DP, Murphy GP, Menck HR (1995). Clinical highlights from the National Cancer Data Base: 1995. CA Cancer J. Clin..

[7] Vallis KA, Tannock IF (2004). Postoperative radiotherapy for breast cancer: Growing evidence for an impact on survival. J. Natl. Cancer Inst..

[8] Nagel PH (2003). Arm morbidity after complete axillary lymph node dissection for breast cancer. Acta. Chir. Belg..

[9] Kwan W (2002). Chronic arm morbidity after curative breast cancer treatment: prevalence and impact on quality of life. J. Clin. Oncol..

[10] Kissin MW (1986). Risk of lymphoedema following the treatment of breast cancer. Br. J. Surg..

[11] Johansson K (2001). Arm lymphoedema, shoulder mobility and muscle strength after breast cancer treatment––a prospective 2-year study. Adv. Physioth.

[12] Delouche G, Bachelot F, Premont M, Kurtz JM (1987). Conservation treatment of early breast cancer: long term results and complications. Int. J. Radiat. Oncol. Biol. Phys..

[13] Chetty U, Jack W, Prescott RJ, Tyler C, Rodger A (2000). Management of the axilla in operable breast cancer treated by breast conservation: a randomized clinical trial. Edinburgh Breast Unit. Br. J. Surg..

[14] Johansen S, Fossa K, Nesvold IL, Malinen E, Fossa SD (2014). Arm and shoulder morbidity following surgery and radiotherapy for breast cancer. Acta Oncol..

[15] Borstad JD (2006). Resting position variables at the shoulder: evidence to support a posture-impairment association. Phys. Ther..

[16] Donatelli, R. A. *Physical therapy of the shoulder*, 3^rd^ edn. (Churchill Livingstone, 2000).

[17] Wedgewood KR, Benson EA (1992). Non-tumour morbidity and mortality after modified radical mastectomy. Ann. Royal College Surg. Engl.

[18] Soulen RL (1997). Musculoskeletal complications of neutron therapy for prostate cancer. Radiat. Oncol. Investig..

[19] Gutman H, Kersz T, Barzilai T, Haddad M, Reiss R (1990). Achievements of physical therapy in patients after modified radical mastectomy compared with quadrantechtomy, axillary dissection and radiation for carcinoma of the breast. Arch. Surg..

[20] Deutsch M (2001). Shoulder and Arm Problems After Radiotherapy for Primary Breast Cancer. Am J Clin Oncol. Apr.

[21] Hidding, J. T. *et al*. Treatment Related Impairments in Arm and Shoulder in Patients with Breast Cancer: A Systematic Review, PLoS One, May 9 (2014). 10.1371/journal.pone.0096748 PMC4016041 24816774

[22] Goyal, S., Buchholz, T. A. & Haffty, B. G. Breast cancer: early stage. In: Halperin, E. C., Wazer, D. E., Perez, C. A., Brady, L. W. editors. Perez and Brady’s principles and practice of radiation oncology. 6th ed. Philadelphia, PA: Lippincott Williams & Wilkins, p. 1044-140 (2013).

[23] Shamley Delva R., Srinanaganathan Ragavan, Weatherall Rosamund, Oskrochi Reza, Watson Marion, Ostlere Simon, Sugden Elaine (2007). Changes in shoulder muscle size and activity following treatment for breast cancer. Breast Cancer Research and Treatment.

[24] Barbara A. SpringerEmail authorEllen LevyCharles McGarveyLucinda A. PfalzerNicole L. StoutLynn H. GerberPeter W. SoballeJerome Danoff, “Pre-operative assessment enables early diagnosis and recovery of shoulder function in patients with breast cancer”, Breast Cancer Research and Treatment, Volume 120, Issue 1, pp 135–147, February (2010). 10.1007/s10549-009-0710-9 PMC2940708 20054643

[25] “How Radiation Therapy Can Affect Different Parts of the Body: If you’re getting radiation therapy to the breast”, https://www.cancer.org/treatment/treatments-and-side-effects/treatment-types/radiation/effects-on-different-parts-of-body.html.

[26] Ingvar C, Johansson K, Albertsson M, Ekdahl C (2001). Arm lymphoedema, shoulder mobility and muscle strength after breast cancer treatment–a prospective 2-year study. Adv. Physiother.

[27] Blomqvist L, Stark B, Engler N, Malm M (2004). Evaluation of arm and shoulder mobility and strength after modified radical mastectomy and radiotherapy. Acta Oncol..

[28] Nesvold IL (2008). Arm and shoulder morbidity in breast cancer patients after breast-conserving therapy versus mastectomy. Acta Oncol..

[29] Contant CM (2000). Morbidity of immediate breast reconstruction (IBR) after mastectomy by a subpectorally placed silicone prosthesis: the adverse effect of radiotherapy. Eur. J. Surg. Oncol..

[30] Richardson ML, Zink-Brody GC, Patten RM, Koh WJ, Conrad EU (1996). MR characterization of post-irradiation soft tissue edema. Skeletal Radiol..

[31] May DA, Good RB, Smith DK, Parsons TW (1997). MR imaging of musculoskeletal tumors and tumor mimickers with intravenous gadolinium: experience with 242 patients. Skeletal Radiol..

[32] Katz J (2005). Risk factors for acute postoperative pain and its persistence following breast cancer surgery: a prospective study. Pain.

[33] Silliman RA (1999). Risk factors for a decline in upper body function following treatment for early stage breast cancer. Breast Cancer Res. Treat..

[34] Wingate L (1985). Efficacy of physical therapy for patients who have undergone mastectomies. A prospective study. Phys. Ther..

[35] Kamath S, Venkatanarasimha N, Walsh MA, Hughes PM (2008). MRI appearance of muscle denervation. Skeletal Radiol..

[36] McMahon CJ, Wu JS, Eisenberg RL (2010). Muscle edema. Am. J. Roentgenol.

